# The role of dexmedetomidine in ARDS: an approach to non-intensive care sedation

**DOI:** 10.3389/fmed.2023.1224242

**Published:** 2023-08-31

**Authors:** Francesca Simioli, Anna Annunziata, Antonietta Coppola, Pasquale Imitazione, Angela Irene Mirizzi, Antonella Marotta, Rossella D’Angelo, Giuseppe Fiorentino

**Affiliations:** Department of Respiratory Pathophysiology and Rehabilitation, Monaldi Hospital, A.O. dei Colli, Naples, Italy

**Keywords:** acute respiratory distress syndrome, non-invasive ventilation, continuous positive airway pressure, pressure support ventilation, high flow nasal cannula, refractory hypoxemia, COVID-19, respiratory drive

## Abstract

**Introduction:**

Severe COVID-19 is a life-threatening condition characterized by complications such as interstitial pneumonia, hypoxic respiratory failure, and acute respiratory distress syndrome (ARDS). Non-pharmacological intervention with mechanical ventilation plays a key role in treating COVID-19-related ARDS but is influenced by a high risk of failure in more severe patients. Dexmedetomidine is a new generation highly selective α2-adrenergic receptor (α2-AR) agonist that provides sedative effects with preservation of respiratory function. The aim of this study is to assess how dexmedetomidine influences gas exchange during non-invasive ventilation (NIV) and high-flow nasal cannula (HFNC) in moderate to severe ARDS caused by COVID-19 in a non-intensive care setting.

**Methods:**

This is a single center retrospective cohort study. We included patients who showed moderate to severe respiratory distress. All included subjects had indication to NIV and were suitable for a non-intensive setting of care. A total of 170 patients were included, divided in a control group (*n* = 71) and a treatment group (DEX group, *n* = 99).

**Results:**

A total of 170 patients were hospitalized for moderate to severe ARDS and COVID-19. The median age was 71 years, 29% females. The median Charlson comorbidity index (CCI) was 2.5. Obesity affected 21% of the study population. The median pO_2_/FiO_2_ was 82 mmHg before treatment. After treatment, the increase of pO_2_/FiO_2_ ratio was clinically and statistically significant in the DEX group compared to the controls (125 mmHg [97–152] versus 94 mmHg [75–122]; ***p < 0.0001). A significative reduction of NIV duration was observed in DEX group (10 [7–16] days vs. 13 [10–17] days; *p < 0.02). Twenty four patients required IMV in control group (*n* = 71) and 16 patients in DEX group (*n* = 99) with a reduction of endotracheal intubation of 62% (OR 0.38; **p < 0.008). A higher incidence of sinus bradycardia was observed in the DEX group.

**Conclusion:**

Dexmedetomidine provides a “calm and arousal” status which allows spontaneous ventilation in awake patients treated with NIV and HFNC. The adjunctive therapy with dexmedetomidine is associated with a higher pO_2_/FiO_2_, lower duration of NIV, and a lower risk of NIV failure. A higher incidence of sinus bradycardia needs to be considered.

## Introduction

COVID-19 is a systemic infection caused by a new coronavirus named severe acute respiratory syndrome coronavirus 2 (SARS-CoV-2), which belongs to the beta family of coronaviruses (1). This virus rapidly spread from Wuhan in China, where it was first isolated, around many countries, causing a pandemic in a few weeks. In January 2020, the director of the world health organization (WHO) declared this event a public health emergency of international concern. By then, all continents had been involved in the pandemic. Italy was early affected by this emergency. Since the first COVID-19 patient in Italy in early 2020, more than 18 million cases and approximately 167,842 deaths have been recorded (2).

The clinical manifestations of SARS-CoV-2 infection widely range between completely asymptomatic cases and critical illness. Severe COVID-19 is a life-threatening condition characterized by complications such as interstitial pneumonia, hypoxic respiratory failure, acute respiratory distress syndrome (ARDS), and pneumomediastinum. Information about the incidence and management of critically ill patients diagnosed with COVID-19 and ARDS are still controversial. Risk factors that have been correlated with a poor prognosis included old age, comorbidities such as systemic hypertension, diabetes mellitus, morbid obesity, chronic pulmonary disease, coronary artery disease, chronic kidney disease, and malignancies (3, 4).

The scientific community faced a significant challenge caused by the pandemic, resulting in several research protocols conducted with therapeutical agents since 2020. A possible indication was tested for a number of active principles, including drugs that modulate the inflammatory response during the infection, such as anti-IL-6R monoclonal antibodies, chloroquine, and hydroxychloroquine, as well as antiviral therapies with various mechanisms of action, for example, neuraminidase inhibitors, inhibitors of DNA and RNA synthesis, lastly a nucleoside analogs inhibitor of the RNA dependent RNA polymerase (RdRp) of coronaviruses. Although some of the drugs showed encouraging results in clinical trials, there is still a major perplexity concerning the severe forms of the disease. Undoubtedly, most of the COVID-19 related deaths are caused by a critical impairment of the lungs and ARDS with alveolar and interstitial damage. The organ damage follows the viral infection and hyper-inflammation phase; finally, it can progress to the point of no return in which reducing the viral load or modulating the inflammation become less relevant targets.

Non-pharmacological intervention with mechanical ventilation plays a key role in treating COVID-19 related ARDS. Non-invasive ventilation (NIV) has been widely accepted in the management of acute respiratory failure, with strong evidence for its benefit in patients with cardiogenic pulmonary edema (5) and acute exacerbations of chronic obstructive pulmonary disease (COPD) (6). Over time NIV has become an established approach for ARDS since it can avoid intubation when used as a first-line intervention in a specific setting with good expertise in ventilation (7). More recently, a review on COVID-19, including 23 manuscripts and 4,776 patients, revealed an appalling NIV failure rate of 47% and an intubation rate of 26.5% (8). At the same time, some authors suggest that high flow nasal cannula (HFNC) can also be successful in selected patients with severe respiratory failure caused by SARS-CoV-2 infection (9). Still the evidence is very poor and debatable.

Dexmedetomidine is a new generation highly selective α2-adrenergic receptor (α2-AR) agonist that provides sedative and analgesic effects, cardiovascular stabilizing effects, and preservation of respiratory function (10). In mechanically ventilated adults, the use of dexmedetomidine, compared to other sedatives, resulted in a lower risk of delirium and a modest reduction in duration of mechanical ventilation and ICU stay but increased the risks of bradycardia and hypotension (11). Scarce evidence is available about the effects of gas exchange during NIV in COVID-19 related ARDS. All the studies about dexmedetomidine are conducted in ICU. There is insufficient evidence about NIV and HFNC in a non-intensive setting of care.

## Aim

The aim of this study is to assess how dexmedetomidine influences gas exchange during NIV and HFNC in moderate to severe ARDS caused by COVID-19 in a non-intensive setting of care. The primary outcomes were the pO_2_/FiO_2_ ratio and the respiratory rate. This study also aims to evaluate the effect of dexmedetomidine on the risk of NIV failure. Secondary outcomes were the intubation rate and the mortality rate.

## Methods

**Figure 1:**
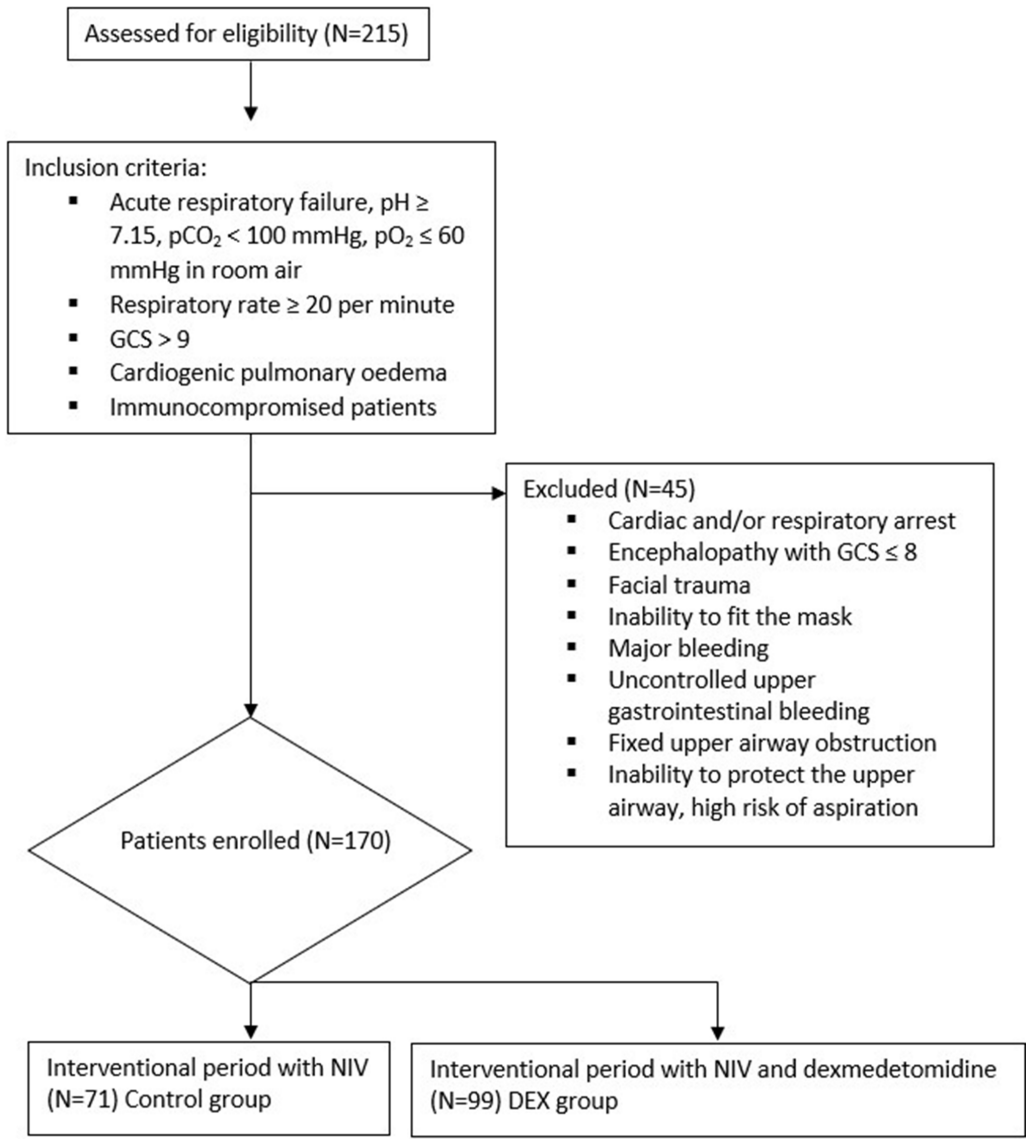
Protocol intervention, inclusion/exclusion criteria.

This is a single center retrospective cohort study. Data were collected from patients who were hospitalized in the sub-intensive therapy respiratory unit at Cotugno Hospital in Naples, Italy, during the COVID-19 pandemic, during 2022, in the post vaccinal era. A total of 215 patients showed acute respiratory failure and moderate to severe respiratory distress at admission. The ratio of arterial oxygen partial pressure to fractional inspired oxygen (pO_2_/FiO_2_ ratio) was 200 or lower. All subjects were tested positive for SARS-CoV-2 by reverse-transcriptase–polymerase-chain-reaction (RT-PCR) assay on a nasopharyngeal swab. All subjects showed interstitial pneumonia with critical extension compatible with severe COVID-19, assessed by a chest’s high-resolution computed tomography (HRCT). A multidisciplinary evaluation at baseline was performed to evaluate the indication to invasive versus non-invasive ventilation; inclusion criteria for NIV were: acute respiratory failure, pH ≥ 7.15, pCO_2_ < 100 mmHg, pO_2_ ≤ 60 mmHg in room air, respiratory rate ≥ 20 per minute, preserved consciousness with Glasgow coma scale (GCS) at least 9, cardiogenic pulmonary oedema, acute hypoxemic respiratory failure in immunocompromised patients. Exclusion criteria for NIV were: cardiac arrest, respiratory arrest, encephalopathy with GCS ≤ 8, facial trauma, inability to fit the mask, major bleeding, uncontrolled upper gastrointestinal bleeding, fixed upper airway obstruction, inability to protect the upper airway, high risk of aspiration. A total of 170 patients had indication to NIV and were suitable for a non-intensive setting of care. A total of 170 patients were included as reported in Figure 1.

At admission, we recorded baseline features: gender, age, BMI and vaccinal status. Concomitant diseases were recorded and the Charlson comorbidity index (CCI) was calculated. CCI is a method of categorizing patients’ comorbidities that predicts the long-term mortality risk (12). The baseline pO_2_/FiO_2_ ratio was determined by blood gas analysis. Ventilation support needed at the time of admission, respiratory rate (RR), and FiO_2_ were recorded. Blood gas analysis was repeated at 2 h after treatment initiation. Subsequently, blood gas analysis was repeated every 6 h. C-reactive protein (CRP) and other markers were tested on blood samples at admission and repeated after treatment initiation in a range of 12–24 h.

The type of respiratory support was chosen based on blood gas analysis, acid–base disorders such as acidosis, the patient’s ventilatory demand and subjective preference, but also the local availability resulted to be determinant during the pandemic. Seventy one subjects received respiratory support and the pharmacological standard of care (control group). Ninety nine subjects received adjunctively dexmedetomidine (DEX group). The administration of sedation depended on the patient’s general status of anxiety and acceptance of ventilation. The two groups were similar considering age, gender, comorbidities, ARDS severity, pO_2_/FiO_2_, respiratory rate, lactates and CRP.

The dexmedetomidine was initiated precociously after NIV administration. The induction of sedation was performed with promazine. The initial dose of dexmedetomidine was 0.3 mcg/kg/h. The maintenance dose of dexmedetomidine ranged between 0.2 and 0.8 mcg/kg/h with a continuous intravenous (IV) infusion. The dose was targeted on the patient’s response, RR, level of consciousness, NIV tolerance and adaptation. Among these, we aimed to have a RR below 25, and a “calm and arousal” status in which the patients were alert and responsive to verbal stimulation. The average dose used during the study was 0.4 mcg/kg/h. During the hospitalization, we recorded what changes in respiratory support and FiO_2_ were needed, as well as the adopted interfaces. We also recorded the duration of NIV in terms of days of treatment before weaning to conventional oxygen therapy (COT). Need for intubation, invasive mechanical ventilation (IVM) and relocation to (ICU) were observed as surrogate outcomes for NIV failure. Mortality was estimated at 30 days from NIV administration. The tolerance to NIV was observed, which included delirium, fighting the ventilator, and pulling the mask. Finally, eventual adverse reactions, especially those on the cardiovascular system, were evaluated.

This study was approved by the local ethics committee of University of Campania “Luigi Vanvitelli” and A.O. dei Colli in accordance with the 1976 Declaration of Helsinki and its later amendments, protocol number: AOC-0020053-2020. All patients were treated accordingly to the local standard of care.

Results are reported as number and percentage for categorical variables and mean and standard deviation (SD) for continuous variables. The mean values of the control group and DEX group were compared using a *t*-test. Confidence interval (CI) was set at 95%, and the significance level was <0.05. The rate of IMV and 30-day mortality rate between the groups were compared using odds ratio (OR).

## Results

A total of 170 patients were hospitalized for moderate to severe ARDS and COVID-19. The median age was 71 years, 29% were females. The median CCI was 2.5. Obesity affected 21% of the study population. All subjects were affected by acute respiratory failure with a median pO_2_/FiO_2_ ratio of 82 mmHg before treatment. The baseline features are described in Table 1. Seventy one patients underwent NIV or HFNC and the standard of care of treatment (control group), 99 patients adjunctively received dexmedetomidine (DEX group). No significant difference was observed at baseline between the two groups, as showed in Table 1.

**Table 1:** Characteristics before treatment.

	Total cohort *n* = 170	Control *n* = 71	DEX *n* = 99	*p*
Age, years	71 (61–80)	70 (60.5–80)	71 (61–79)	0.56
Female	49 (29%)	19 (27%)	30 (30%)	
Charlson comorbidity index	2.5 (2–4)	2.5 (1.5–4)	2.75 (2–5)	0.24
Obesity (BMI > 30)	36 (21.17%)	17 (23.9%)	19 (19%)	0.20
pO_2_/FiO_2_, mmHg	82 (69–108)	82 (72–120)	82 (68–105)	0.14
Respiratory rate (RR), pm	30 (28–34)	30 (28–33)	30 (28–34)	0.33
FiO_2_, %	80 (60–95)	80 (60–95)	80 (66–95)	0.45
Lactate, mmol/L	1 (1–5)	2 (1–5)	1 (1–5)	0.23
C-reactive protein (CRP), mg/dL	6 (3–10)	6 (3–9.5)	6 (3–10)	0.32
SARS-CoV-2 vaccine	110 (64.7%)	46 (64.8%)	64 (64.6%)	

Results are expressed as median and IQR or number and percentage.

The HFNC was administered with a mean flow of 60 L/min. The mean positive end expiratory pressure (PEEP) in the CPAP mode was 8 cm H₂O. The mean pressure support (PS) and expiratory positive airway pressure (EPAP) in the NIV—PSV mode was 12 and 8 cm H₂O, respectively.

After treatment, the pO_2_/FiO_2_ ratio clearly increased in both groups compared to baseline (106 vs. 82 mmHg in the total cohort); the increase was clinically and statistically significant in the DEX group compared to the controls (125 mmHg [97–152] vs. 94 mmHg [75–122]; 95% CI 16.46–45.54; ***p < 0.0001). The RR improved at the same time, without a significant difference between the DEX and the control group (24 [20–26] pm vs. 26 [22–26] pm; p = ns). No significant difference was observed on other biochemical tests, including CRP (5 vs. 5 mg/dL; p = ns). The results are reported in Table 2.

**Table 2:** Characteristics after treatment.

	Total cohort (*n* = 170)	Control (*n* = 71)	DEX (*n* = 99)	*p*
Respiratory support				
- HFNC	83 (49%)	38 (53%)	45 (46%)	
- CPAP	41 (24%)	16 (23%)	25 (25%)	
- NIV	46 (27%)	17 (24%)	29 (29%)	
pO_2_/FiO_2_, mmHg	106 (90–148)	94 (75–122)	125 (97–152)	0.0001***
RR, pm	24 (20–26)	26 (22–28)	24 (20–26)	0.07
Lactate, mmol/L	2 (1–5)	2 (1–5)	2 (1–6)	0.83
CRP, mg/dL	5 (2–8)	5 (2–8)	5 (3–8)	0.73
NIV duration, days	11 (8–16)	13 (10–17)	10 (7–16)	0.02*
IMV, %	40 (23.5%)	24 (33.8%)	16 (16%)	0.008**
30 days mortality, %	30 (17.6%)	16 (25.4%)	12 (12.1%)	0.07

Median and IQR or number and percentage. *p ≤ 0.05; **p ≤ 0.01; ***p ≤ 0.001.

A significative reduction of NIV duration was observed in DEX group (10 [7–16] days vs. 13 [10–17] days; 95% CI −5.53 to −0.47; *p < 0.02). Twenty four patients required IMV in control group (*n* = 71) and 16 patients in DEX group (*n* = 99) with a reduction of endotracheal intubation of 62% (OR 0.38; 95% CI 0.18–0.78; **p < 0.008). The 30-days mortality was calculated among non-invasively ventilated patients, 16 deaths were recorded in control group and 12 in DEX group with a non-significant reduction of the risk of death (OR 0.47; 95% CI 0.21–1.07; *p* < 0.07).

**Figure 2:**
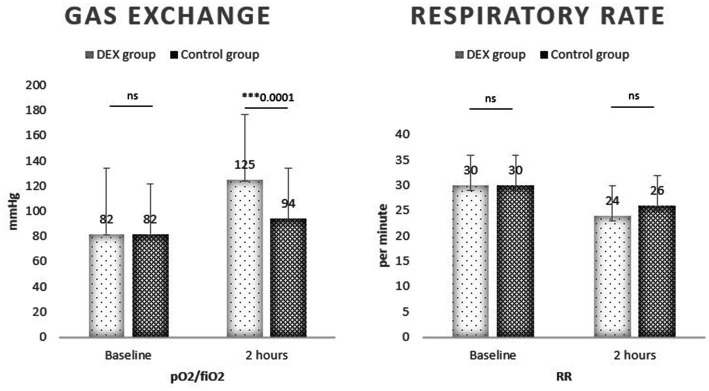
pO_2_/FiO_2_ improved in DEX group; respiratory rate was comparable.

**Figure 3:**
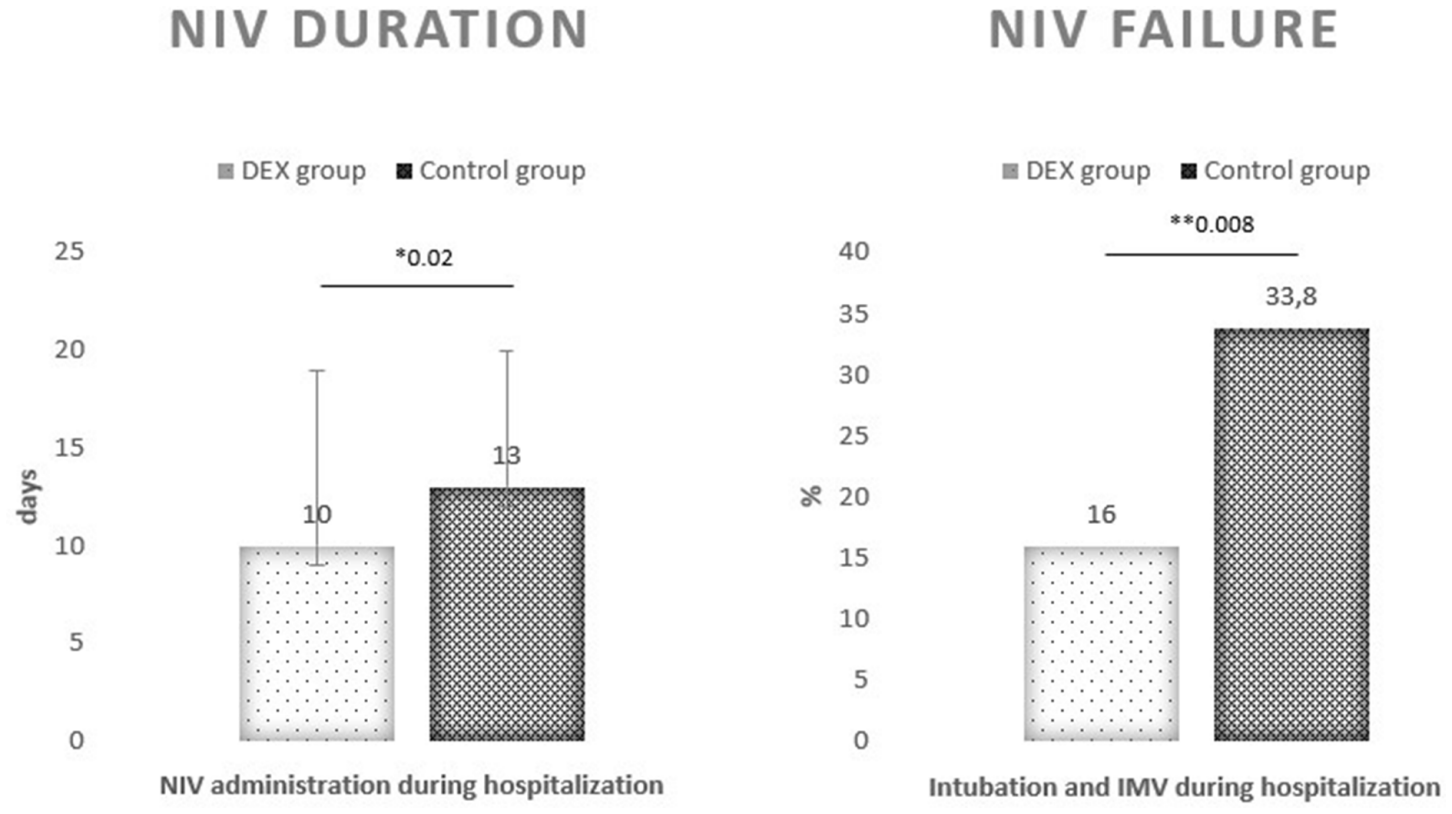
Non-invasive ventilation was needed for a shorter time in DEX group compared to controls. Invasive mechanical ventilation (IMV) rate was lower in DEX group.

Outcomes on gas exchange and NIV failure are reported in Figures 2, 3.

Hypotension was observed in 11% of the total population during hospitalization, the incidence was similar between DEX and the control group (12% vs. 10%). Bradycardia occurred in 25% of cases and was frequently associated with dexmedetomidine administration (30%). A minority of those effects were considered severe and led to dexmedetomidine discontinuation. Tachyarrhythmia was observed in 4.7% of the population with a comparable incidence between the groups.

Intolerance to NIV was very frequent in our study population (33.5%). Delirium was observed more frequently in the control group than DEX (22% vs. 16%), but no significant difference was observed (OR 0.67; 95% CI 0.35–1.26; *p* = 0.22) (Table 3).

**Table 3:** Concomitant observations and adverse drug reactions.

	Total cohort	Control	DEX
Hypotension	19 (11.2%)	7 (10%)	12 (12%)
Bradycardia	45 (25.3%)	13 (18.3%)	30 (30%)
Tachyarrhythmia	8 (4.7%)	3 (4.2%)	5 (5%)
NIV intolerance	64 (33.5%)	26 (36.6%)	31 (31%)
Delirium	31 (18.23%)	16 (22.53%)	15 (16%)

## Discussion

The main purpose of the study was to investigate the effects of dexmedetomidine on gas exchange during NIV and HFNC in moderate to severe ARDS caused by COVID-19 in a non-intensive setting of care. Dexmedetomidine is an α2-adrenoceptor agonist with sedative, anxiolytic, sympatholytic, and analgesic-sparing effects, and minimal depression of respiratory function. It is potent and highly selective for α2-receptors with an α2:α1 ratio of 1,620:1. Dexmedetomidine exerts its hypnotic action through activation of central pre- and postsynaptic α2-receptors in the locus coeruleus, thereby inducting a state of unconsciousness similar to natural sleep, with the unique aspect that patients remain easily rousable and cooperative. Dexmedetomidine is rapidly distributed and is mainly hepatically metabolized into inactive metabolites by glucuronidation and hydroxylation. A high inter-individual variability in dexmedetomidine pharmacokinetics has been described, especially in the intensive care unit population. In recent years, multiple pharmacokinetic non-compartmental analyses as well as population pharmacokinetic studies have been performed. Body size, hepatic impairment, and presumably plasma albumin and cardiac output have a significant impact on dexmedetomidine pharmacokinetics. As central α1-adrenoceptor activation counteracts the sedative α2 effects, dexmedetomidine is a more potent sedative than clonidine. An important feature of dexmedetomidine-based sedation is that patients remain easily rousable. This aspect, combined with the minimal influence on respiration, makes dexmedetomidine an interesting alternative sedative in many procedures, such as awake craniotomies and conscious sedation of non-intubated patients prior to and/or during surgical and other procedures (13). Despite this, dexmedetomidine use is actually confined to intensive care. The aim of the study was to test its use in a non-intensive setting of care.

As the primary outcome, we measured the pO_2_/FiO_2_ ratio, which significantly improved in the DEX group. This observation suggests a positive effect on oxygenation without the risk of significant respiratory depression for all types of respiratory support; in fact, 52% of the patients enrolled received HFNC, 46% in the DEX group. The respiratory rate instead was similar in the two groups, thus suggesting that the pattern of breathing was guaranteed by the respiratory support and the patient’s effort itself even during sedation. COVID-19 related ARDS is characterized by high work of breathing and ventilatory demand, and this has been associated with self-inflicted lung injury (14). Patients in the DEX group were “calm and arousal” and maintained spontaneous ventilation. We can speculate that dexmedetomidine contributed to matching the patient’s ventilatory demand, avoiding unregulated and prolonged efforts. We included patients with moderate to severe ARDS with pO_2_/FiO_2_ ratio < 200, but they were mostly severe considering the mean pO_2_/FiO_2_ ratio of 82 at baseline. We decided to include all types of respiratory therapy, also considering that during pandemic the initial therapy was partially influenced by local availability beyond clinical considerations or laboratory features. Among the overall population, 49% received HFNC, 24% CPAP, and 27% NIV (pressure support). The average needed FiO_2_ was 0.80 in both groups. The wide heterogeneity of respiratory therapies may represent a limit, and it was considered as a confounding factor during the data analysis process. Nevertheless, we chose to include all possible respiratory therapies in relation to real life practice, and also because we aimed to test the adaption to ventilation besides the physiological effects of different therapies. Many patients worsen not because receiving inappropriate therapy, but they may not benefit of them because of agitation and asynchronies, leading to an increased work of breathing, fatigue and lower oxygenations despite alveolar recruitment. This study also aims to evaluate the effect of dexmedetomidine on the risk of NIV failure. The treatment group showed a smaller duration of ventilation (10 days) and a smaller intubation rate (16%). According to the literature, dexmedetomidine is a strategy to improve NIV comfort; it reduces the need for intubation and mechanical ventilation in adults with acute respiratory failure in ICU (15). Our observation brings dexmedetomidine outside ICU, and it has to be considered a safe adjunctive therapy for COVID-19 with associated acute respiratory failure for its effects on gas exchange and comfort. NIV intolerance was unsurprisingly common in our population and was similarly observed in both groups at baseline (33.5%). As a matter of fact, the adhesion to therapy and cooperation, which is fundamental in awake patients, clinically improved with continuous dexmedetomidine infusion. Delirium was as common as data reported by ICU-based studies. Nevertheless, we observed a significant reduction of delirium in DEX group compared to no sedation (16% vs. 22%). This finding likely explains the lower need for IMV and the lower mortality overall.

A large observational study reported that NIV is used to treat ARDS in ICU, but IMV should be adopted for severe ARDS with pO_2_/FiO_2_ ratio lower than 150 mmHg (16). NIV strategies appear safe in mild-to-moderate hypoxemia, whereas HFNC and helmet represent the most promising techniques for first-line treatment of ARDS, but some evidence support IMV for severe cases in order to avoid delayed intubation and increased mortality (17). On the contrary, our study promotes the use of a more conservative approach even in severe ARDS. Early initiation of non-invasive respiratory support and dexmedetomidine can implement oxygenation and match ventilatory demand in spontaneously breathing awake patients, avoiding vigorous inspiratory effort. A more homogeneous distribution of the tidal volume and the alveolar recruitment by a positive end-expiratory pressure protect from lung strain and diaphragm dysfunction. However, spontaneous breathing in patients with lung injury carries the risk of delayed intubation and additional lung damage during the treatment. The phenomenon is described as patient self-inflicted lung injury (P-SILI). This study suggests that the adjunctive administration of dexmedetomidine contributes to contrasting an abnormal pattern of breathing; additionally, a conscious and cooperative sedation, such as what is obtained with this drug, relieves the psychological component of dyspnea and facilitates tolerance. Finally, a minor probability of NIV failure implies preventing intubation, related complications and mortality.

The management of ARDS is very challenging. But respiratory distress does not represent an absolute indication to IMV. Prolonged ventilation means prolonged hospitalization, and increases the risk of tracheostomy, oral feeding, muscle weakness and other complications such as multi-drug resistant infections. This is a very remarkable point since severe COVID-19 causes a persistent dysregulation of immunity. This study remarks the fundamental role of NIV in ARDS. Severe patients can safely undergo NIV in non-intensive care of setting for ARDS, provided with consciousness and with a precise monitoring of failure signals. The sedation with dexmedetomidine modifies the success rate of NIV and is potentially useful in clinical management and decision making.

Regarding the adverse events, liver and kidney function were not involved in relevant consequences even with prolonged infusion of dexmedetomidine. The most common effects are on the cardiovascular system. Hypotension was observed in 12% of DEX group but was as frequent as in the total cohort. Bradycardia was significantly associated with dexmedetomidine (30% of DEX group). The bradycardia was considered serious and the treatment was stopped in 10% of cases. Since bradycardia was observed in 18% of the control group, it is impossible to exclude that other factors or drugs played a part in determining this adverse event.

## Limitations

This study has several limitations. First, this is not a multicentric protocol, the data collection was conducted in only one hospital in Naples, even though in one of the most populous cities in Italy. Secondly, the total number of subjects is small. Particularly we collected data from different subsequent waves of COVID-19 regardless for the predominance of different variants and subvariants of the virus. Thirdly, the follow up time is relatively short compared to the natural history of the disease, but it was targeted on the specific outcome gas exchange which needs to be tested on the short run to correctly manage ARDS. Finally, no definitive recommendations can be made about a single approach but a dynamic evaluation of the single case is to be endorsed to promptly detect the need for endotracheal intubation.

## Conclusion

Dexmedetomidine provides a “calm and arousal” status which allows spontaneous ventilation in awake patients treated with NIV and HFNC for COVID-19 related ARDS. The adjunctive therapy with dexmedetomidine is associated with a higher pO_2_/FiO_2_, lower duration of NIV, and a lower risk of NIV failure. a continuous monitoring of vital signs is recommended to promptly detect anomalies. A higher incidence of sinus bradycardia needs to be considered.

## Data availability statement

The original contributions presented in the study are included in the article/supplementary material, further inquiries can be directed to the corresponding author.

## Ethics statement

The studies involving human participants were reviewed and approved by committee of the University of Campania “Luigi Vanvitelli” and A.O. dei Colli. The patients/participants provided their written informed consent to participate in this study.

## Author contributions

FS, AA, and GF: conceptualization and methodology. FS: software and formal analysis. AC, PI, and AM: validation. AC, PI, AIM, RD’A, and AM: investigation. AM, AC, and AIM: resources. PI, AIM, RD’A, and AM: data curation. FS, AC, and AIM: writing. AA, GF, and PI: visualization. AA, GF, and AM: supervision. FS, RD’A, AIM, and PI: project administration. All authors contributed to the article and approved the submitted version.

## Conflict of interest

The authors declare that the research was conducted in the absence of any commercial or financial relationships that could be construed as a potential conflict of interest.

## Publisher’s note

All claims expressed in this article are solely those of the authors and do not necessarily represent those of their affiliated organizations, or those of the publisher, the editors and the reviewers. Any product that may be evaluated in this article, or claim that may be made by its manufacturer, is not guaranteed or endorsed by the publisher.
